# Efficacy and Safety Evaluation of Sintilimab for Cancer Treatment: A Systematic Review and Meta-analysis of Randomized Controlled Trials

**DOI:** 10.3389/fphar.2022.895187

**Published:** 2022-04-28

**Authors:** Ziqi Ye, Wenchao Yang, Bixia Xuan, Xiaofang Li, Jiana He, Haiyan Si, Wenhua Ma

**Affiliations:** ^1^ Department of Clinical Pharmacy, The First Affiliated Hospital, Zhejiang University School of Medicine, Hangzhou, China; ^2^ Department of Pharmacy, Traditional Chinese Medical Hospital of Zhuji, Zhuji, China; ^3^ Department of Gastroenterology, Traditional Chinese Medical Hospital of Zhuji, Zhuji, China

**Keywords:** Sintilimab, cancer, overall survival, progression free survival, safety

## Abstract

**Objective:** Meta analysis was used to explore the efficacy and safety of Sintilimab in the treatment of cancer.

**Methods:** The databases of CNKI, VIP, Wanfang Data, PubMed, ScienceDirect, the Cochrane Library and EMBASE were searched by computer to collect the randomized controlled trials published as of March 2022. The retrieval work was completed by two researchers alone. They screened the literature and extracted the data according to the nanodischarge standard, using Revman 5.4 software. The included studies were statistically analyzed.

**Results:** Six RCTs were included in this study, including 1,048 cases of Sintilimab and 711 cases of other anticancer drugs. Compared with the control group, the overall survival (HR = 1.64, 95% CI: 1.35–1.99, *p* < 0.00001) and progression free survival (HR = 1.89, 95% CI: 1.59–2.25, *p* < 0.00001) of cancer treated with Sintilimab were longer and more effective. Moreover, the risk ratio of any grade of adverse reactions (HR = 0.87, 95% CI: 0.74–1.03, *p* = 0.11) and above grade III adverse reactions (HR = 0.84, 95% CI: 0.67–1.06, *p* = 0.14) in the treatment of cancer with Sintilimab was lower and the safety was better.

**Conclusion:** Compared with non-Sintilimab group, Sintilimab treatment can improve the clinical efficacy of tumor patients and has a lower incidence of adverse reactions. This treatment may be a promising treatment for cancer patients.

**Systematic Review Registration:** (website), identifier (registration number).

## Introduction

The global epidemiological survey shows that cancer has high incidence rate and mortality rate. Clinically, traditional dual drug chemotherapy including platinum, paclitaxel and adriamycin has always been the standard first-line therapy for cancer patients (Wei et al., 2020). In recent years, immune checkpoint inhibitors (ICIs) have been more and more widely used in clinic. Multiple ICIs have been proved to improve the survival rate of cancer patients, but the high price and high medical cost are still the main obstacles for Chinese cancer patients to obtain these treatments. For these reasons, the successful development of ICIs made in China has greatly reduced the economic burden of patients and benefited more patients. Sintilimab was officially approved by the State Drug Administration in December 2018 and was listed in the national medical insurance catalogue in November 2019 (Wang et al., 2019). Sintilimab is an inhibitor of recombinant human immunoglobulin G-type programmed death protein-1 (PD-1). It has been approved for the treatment of recurrent or refractory classical Hodgkin’s lymphoma since December 2018. At present, it has carried out extensive clinical trials in solid tumors such as lung cancer, liver cancer, gastric cancer and esophageal cancer (Huang et al., 2022). This study systematically evaluated the efficacy and safety of Sintilimab in the treatment of cancer, in order to provide reference basis for clinical treatment.

## Materials and Methods

### Search Strategy and Study Selection

We searched CNKI, VIP, Wanfang Data, PubMed, ScienceDirect, the Cochrane Library, EMBASE and other databases by computer. In addition, we also searched the references and meeting minutes included in the study to supplement and obtain relevant materials. Chinese key words: Sintilimab, cancer, randomized controlled trial English key words: Sintilimab, IBI308, IBI-308, cancer, randomized controlled trials, RCTs. The search time is up to March 2022.

Inclusion criteria: ① the type of study is RCTs; ② The subjects were patients diagnosed with cancer by clinicopathological examination; ③ Intervention measures: patients in the experimental group were treated with Sintilimab, and patients in the control group were treated with non Sintilimab (the treatment without Sintilimab); ④ The primary outcome measures were overall survival (OS) and progression free survival (PFS). The secondary outcome measures were adverse reactions at any level and adverse reactions above grade 3.

Exclusion criteria: ① repeatedly published literature; ② Documents that cannot obtain original data or contact the author to obtain the original text; ③ Abstract, review, meta-analysis, case report and animal experiment; ④ Non Chinese and English literature.

### Bias Risk Assessment and Quality Assessment

The Newcastle Ottawa scale (NOS) was used to evaluate the quality of the included study (Bylicki et al., 2018), according to the following: 1) whether it is representative; 2) Determination of blind method; 3) Whether the random method is determined; 4) Completeness of outcome events; 5) Comparability of included studies; 6) Evaluation of outcome events; 7) Whether there is follow-up; 8) Follow up integrity. High quality literature is seven to nine points, general quality literature is four to six points, and low quality literature is three points or lower. Two reviewers independently extracted data according to the specified selection criteria. Differences of opinion are resolved through discussion between authors or by obtaining opinions from a third evaluator.

### Data Extraction

Two researchers independently screened the literature, extracted the data and cross checked. In case of any difference, it shall be settled through discussion or negotiation with a third party. During literature screening, first read the title. After excluding the obviously irrelevant literature, further read the abstract and full text to determine whether to be included. If necessary, contact the original study author by email or telephone to obtain information that is not determined but very important to this study. Data extraction contents include: ① basic information of the included study: first author, year of publication, sample size, outcome indicators and outcome measurement data.

### Statistical Analysis

Data were processed through Revman5.4 software. Relative risk ratios (RR) and 95% confidence interval (95% CI) were used as effect indexes for counting data, and the difference was statistically significant (*p* < 0.05). I^2^ is used to evaluate the heterogeneity. If the heterogeneity test result I^2^ is less than 50%, it means that there is no statistical heterogeneity among the research results, and the fixed effect model is used; If the heterogeneity test result I^2^ > 50%, analyze the source of heterogeneity. If the heterogeneity still exists, select the random effect model to estimate the combined effect.

## Results

### Literature Search and Characteristics of Included Studies

**FIGURE 1:**
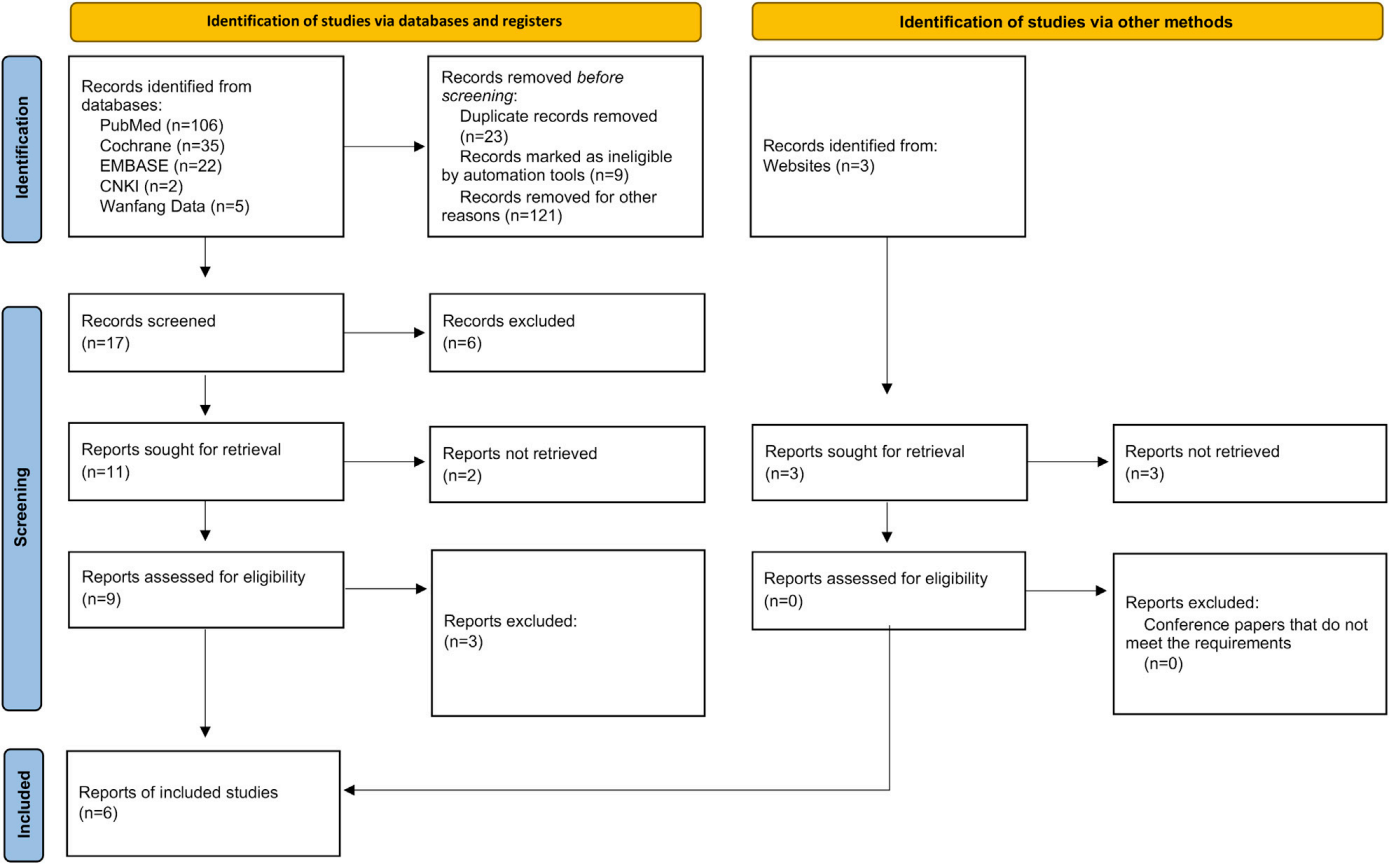
Literature screening process and results.

We searched 170 literatures (including 106 in PubMed, 35 in Cochrane, 22 in Embase, two in CNKI and five in Wangfang Data) and three conference papers by computer, and selected 17 according to the title and abstract. After full-text analysis and evaluation, we excluded 11 literatures with abnormal data, incomplete information or unavailable due to non comparative research, and finally included 6 (Shi et al., 2019; Yang et al., 2020; Ren et al., 2021; Zhou et al., 2021; Lin et al., 2022; Xu et al., 2022) literatures for systematic evaluation and meta-analysis. The process of literature screening is shown in Figure 1. There were 1,048 patients in the experimental group and 711 patients in the control group. The six literatures are of high quality and their basic characteristics and main evaluation indicators were shown in Table 1.

**TABLE 1:** Basic characteristics of included studies and main evaluation indicators (Shi et al., 2019; Yang et al., 2020; Ren et al., 2021; Zhou et al., 2021; Lin et al., 2022; Xu et al., 2022).

First author	Year	Clinical trial number	Phase	Type of cancer	NO. of patients with sintilimab	NO. of patients with non-sintilimab	Gender		Average age	HR for OS (95%CI)	*p*-Value for OS	HR for PFS (95%CI)	*p*-Value for PFS	Quality
							Sintilimab (M/F)	Non-sintilimab (M/F)						
Yunpeng Yang Lin et al. (2022)	2020	NCT03607539	III	NSCLC	266	131	204/62	99/32	61	0.609 (0.4,0.926)	0.01921	0.482 (0.362,0.643)	<0.00001	7
Zhenggang Ren Ren et al. (2021)	2021	NCT03794440	II-III	HC	380	191	334/46	171/20	53–54	0.57 (0.43,0.75)	<0.0001	0.56 (0.46,0.70)	<0.0001	7
Caicun Zhou Shi et al. (2019)	2021	NCT03629925	III	NSCLC	179	178	163/16	164/14	62–64	NA	NA	0.536 (0.422,0.681)	<0.00001	8
Xinqing Lin Xu et al. (2022)	2021	NCT03629925	III	NSCLC	32	20	29/3	17/3	58–63	0.62 (0.28,1.36)	0.23	0.61 (0.30,1.25)	0.18	9
Yuankai Shi Yang et al. (2020)	2019	NCT03114683	II	Hodgkin lymphoma	96	96	56/40	56/40	33	NA	NA	NA	NA	7
Jianming Xu Zhou et al. (2021)	2022	NCT03116152	II	ESSC	95	95	88/7	84/11	60	0.70 (0.50,0.97)	0.032	NA	NA	8

Note: HC, hepatocellular carcinoma; ESSC, Esophageal squamous-cell carcinoma; HR, hazard ratio; NA, Not available; PFS, progression free survival; OS, overall survival; M, male; F, female.

### Meta Analysis Results of Efficacy

**FIGURE 2:**
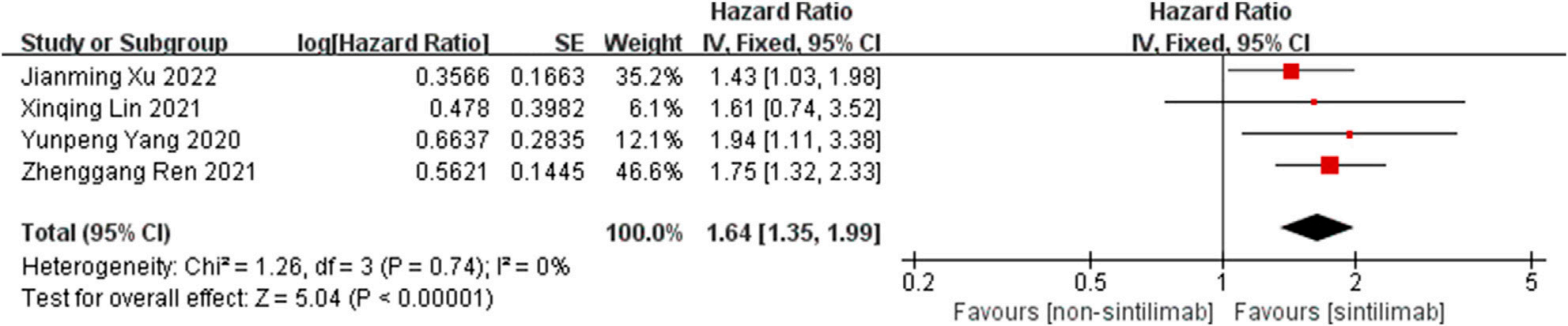
Meta-analysis results of OS between Sintilimab group and non-Sintilimab group.

Comparison of OS: four studies can obtain the OS data of cancer patients treated with Sintilimab. For heterogeneity analysis, I^2^ = 0%, *p* = 0.74. There is no statistical heterogeneity among the studies. The fixed effect model is used for analysis. The results showed that HR = 1.64 (95% CI = 1.35–1.99, *p* < 0.00001), suggesting that Sintilimab can significantly improve the OS of cancer patients, as shown in Figure 2.

**FIGURE 3:**
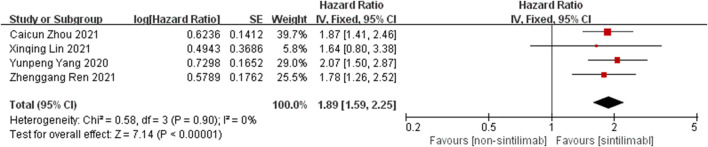
Meta-analysis results of PFS between Sintilimab group and non-Sintilimab group.

Comparison of PFS: the PFS data of cancer patients treated with Sintilimab can be obtained from four studies. For heterogeneity analysis, I^2^ = 0%, *p* = 0.90. There is no statistical heterogeneity among the studies. The fixed effect model is used for analysis. The results showed that HR = 1.89 (95% CI = 1.59–2.55, *p* < 0.00001), suggesting that Sintilimab can significantly prolong PFS in cancer patients, as shown in Figure 3.

### Meta Analysis Results of Safety

**FIGURE 4:**
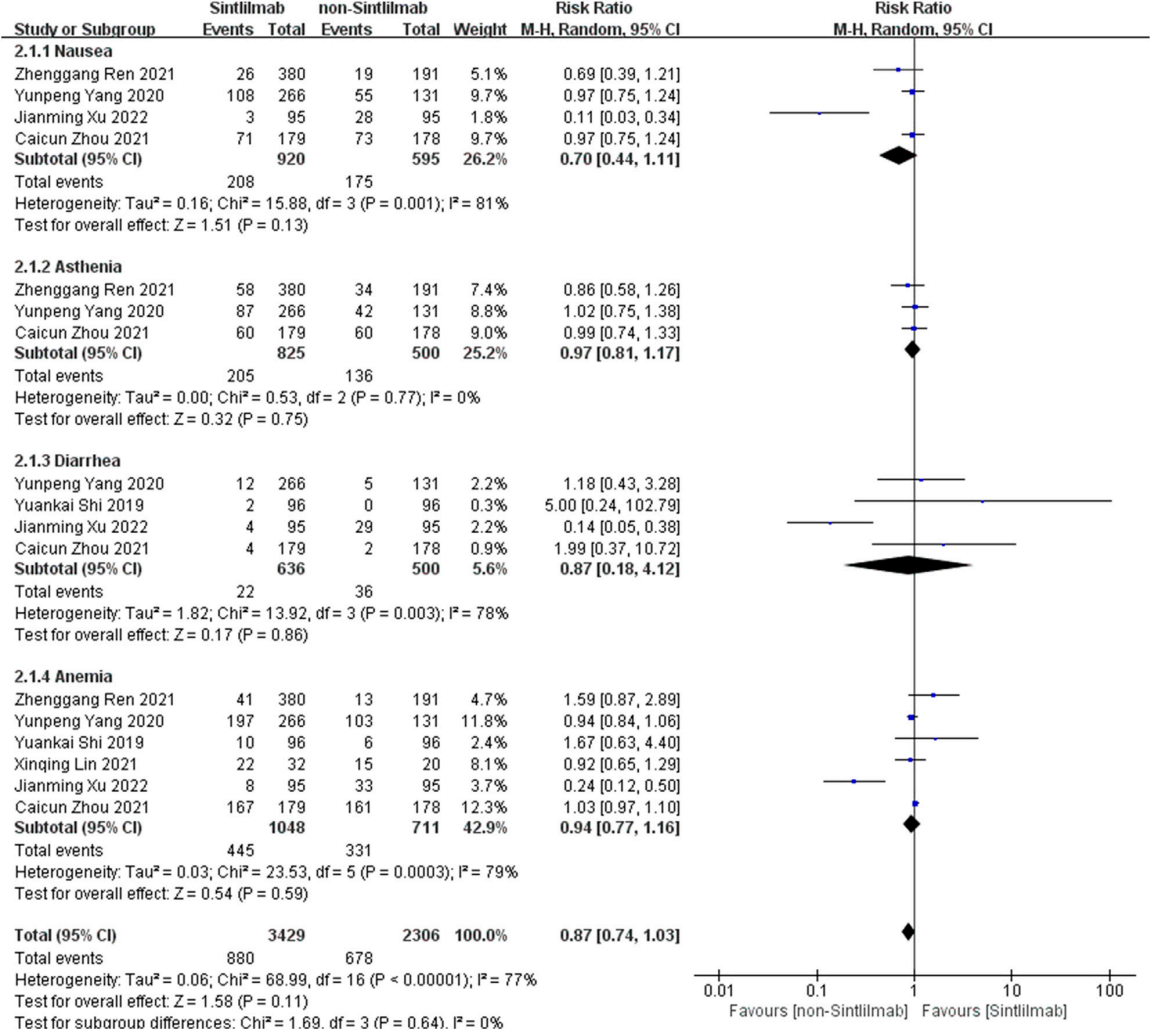
Meta-analysis results of adverse reactions of any grade between Sintilimab group and non-Sintilimab group.

Comparison of adverse reactions at any level: Six studies can obtain the data of any level of adverse reactions (including nausea, ashenia, diarrhea and anemia) of cancer patients treated with Sintilimab. For heterogeneity analysis, I^2^ = 77%, *p* < 0.00001. There is statistical heterogeneity among studies, which is analyzed by random effect model. The results showed that HR = 0.87 (95% CI = 0.74–1.03, *p* = 0.11), suggesting that the incidence of any level of adverse reactions in patients with Sintilimab was low, but there was no significant difference in the results, as shown in Figure 4.

**FIGURE 5:**
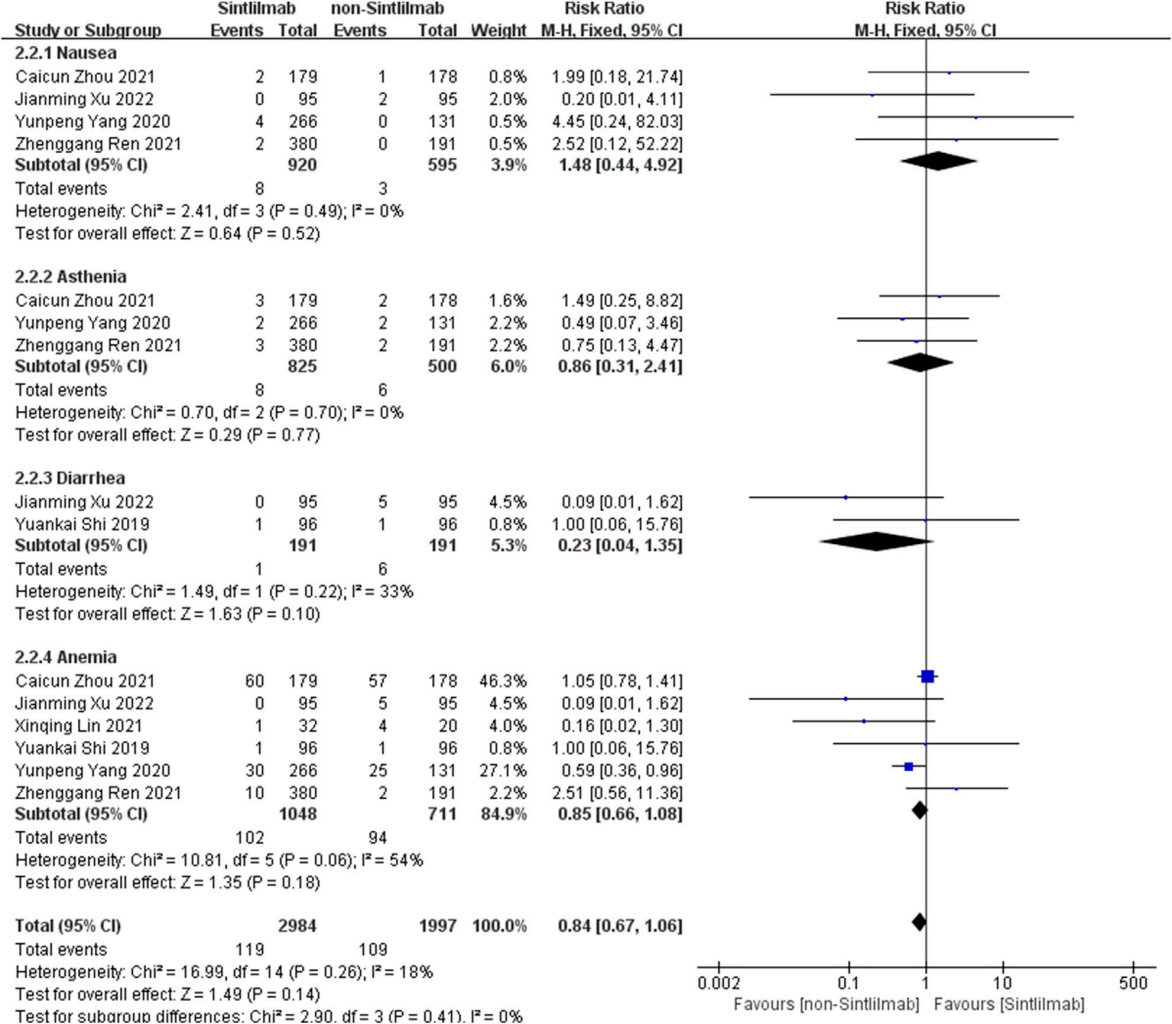
Meta-analysis results of adverse reactions above grade 3 between Sintilimab group and non-Sintilimab group.

Comparison of adverse reactions above grade III: Six studies can obtain the data of more than grade III adverse reactions (including nausea, ashenia, diarrhea and anemia) of cancer patients treated with Sintilimab. The heterogeneity analysis is carried out, with I^2^ = 18%, *p* = 0.26. There is no statistical heterogeneity among the studies. The fixed effect model is used for analysis. The results showed that HR = 0.84 (95% CI = 0.67–1.06, *p* = 0.14), suggesting that the incidence of grade III and above adverse reactions in patients with Sintilimab was low, but there was no significant difference in the results, as shown in Figure 5.

### Publication Bias Assessment and Sensitivity Analysis

**FIGURE 6:**
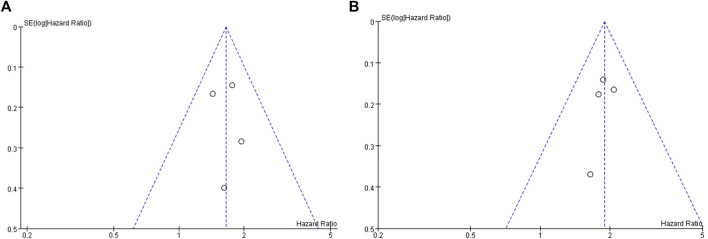
Inverted funnel plot of OS (A) and PFS (B).

The publication bias assessment of this study was performed only in OS and PFS. The funnel plot is symmetrical, indicating no significant publication bias (Figure 6). Sensitivity analysis was conducted on the results, and meta-analysis was conducted by ignoring each study in turn. No significant changes were found in the results, indicating that the results of this study are stable.

## Discussion

Sintilimab is a monoclonal antibody against programmed cell death protein 1. It can block the interaction between PD-1 and its ligand and help T cells restore their anti-tumor effect. In 2018, Sintilimab has been approved by the State Administration of medicine of China for the treatment of patients with recurrent or refractory classical Hodgkin’s lymphoma. In recent years, a large number of studies have reported the anti-tumor effect of Sindilimab. In general, Sintilimab has similar anti-tumor effect and better safety compared with other ICIs (such as Nivolumab and Pembrolizumab) in Hodgkin’s lymphoma, natural killer T-cell lymphoma and advanced non-small cell lung cancer (Zhang et al., 2020).

In phase three clinical trials, the combination of ICIs and chemotherapy is rapidly developing into a first-line treatment for many cancers. Paz-Ares et al. (Paz-Ares et al., 2018) showed that Pembrolizumab combined chemotherapy (carboplatin and paclitaxel or paclitaxel) significantly prolonged OS (median, 15.9 vs. 11.3 months, HR = 0.64, *p* < 0.001) and PFS (6.4 vs. 4.8 months, HR = 0.56, *p* < 0.001) compared with chemotherapy alone. Martin Reck et al. (Reck et al., 2016) found that the median progression free survival of Pembrolizumab combined with chemotherapy group was significantly prolonged, 10.3 vs. 6.0 months. The 6-month overall survival rate in the Pembrolizumab combined chemotherapy group was estimated to be 80.2% vs. 72.4%. The above suggests that the overall survival and progression free survival of cancer patients treated with ICIs combined with chemotherapy have been significantly improved. As an ICIs, Sintilimab has been used in combination with chemotherapeutic drugs in the treatment of cancer patients. For example, this randomized, open label, multicenter phase two trial of Jianming Xu et al. (Ren et al., 2021) evaluated the comparison of PD-1 inhibitor Sintilimab with chemotherapy in patients with esophageal squamous cell carcinoma after first-line chemotherapy. Compared with the chemotherapy group, the median OS in the Sintilimab group was significantly improved (median OS 7.2 vs. 6.2 months; *p* = 0.032; HR = 0.70; 95% CI, 0.50–0.97). The incidence of grade 3–5 treatment-related adverse events in the Sintilimab group was lower than that in the chemotherapy group (20.2% and 39.1%, respectively).

In this meta-analysis, we evaluated the efficacy of Sintilimab in cancer patients and selected OS and PFS as the primary outcomes. The results showed that in terms of effectiveness, Sintilimab improved the HR of OS and PFS, indicating that patients receiving immunotherapy had better OS and PFS than patients receiving ordinary chemotherapy. In terms of safety, the risk ratio of adverse reactions at any level and above in the Sintilimab treatment group is lower than that in the control group. Although there is no significant difference in the results, it also suggests that the safety of Sintilimab treatment is higher than that of ordinary chemotherapy drugs, and it is not easy to produce more common typical adverse reactions (nausea, ashenia, diarrhea, anemia). According to the above results suggest that in the process of clinical practice, especially for non-small cell lung cancer, liver cancer, non-hodgkin’s lymphoma, such as cancer patients during chemotherapy, Sintilimab can be used as the preferred drug resistance. It can not only bring a higher response rate, also can prolong the overall survival and disease progression, and caused most of the adverse reaction of one to two levels. With a long-lasting therapeutic response and tolerable toxicity, Sintilimab has shown promising efficacy overall.

It has to be said that this study also has some limitations: ① after systematic retrieval and screening, only six limited literatures were included for systematic evaluation and meta-analysis, resulting in a small sample size; ② The heterogeneity of individual statistical results may affect the credibility of the research results; ③ Different cancer types in different studies may increase heterogeneity and affect the reliability of results. However, in the study, in order to better reduce the above bias, when implementing retrieval and data consolidation, this study should be scientifically and objectively reported according to the Newcastle Ottawa scale as much as possible.

In conclusion, compared with non-Sintilimab group, Sintilimab can prolong the OS and PFS of patients in the treatment of cancer, with better clinical efficacy and high safety. Sintilimab may be a promising treatment for cancer patients.

## Data Availability

The original contributions presented in the study are included in the article/Supplementary Material, further inquiries can be directed to the corresponding author.
